# Fine‐needle aspiration cytology diagnosis of aspergilloma – A case report

**DOI:** 10.1002/ccr3.5826

**Published:** 2022-05-12

**Authors:** Dilasma Ghartimagar, Manish Kiran Shrestha, Arnab Ghosh, Dipesh Upreti

**Affiliations:** ^1^ 92963 Department of Pathology Manipal College of Medical Sciences Pokhara Nepal; ^2^ Department of Radiology Pokhara Academy of Health Sciences Pokhara Nepal; ^3^ 592234 Department of Pathology Manipal Tata Medical College Jamshedpur India; ^4^ 92963 Manipal College of Medical Sciences Pokhara Nepal

**Keywords:** aspergilloma, fine‐needle aspiration cytology, lung

## Abstract

Fine‐needle aspiration cytology, a simple and inexpensive technique can aid in early diagnosis of aspergilloma. Here, we present a case of 55‐years‐old female with a past history of pulmonary tuberculosis and a right‐lung cavitary lesion, diagnosed as aspergilloma.

## 1 INTRODUCTION

Aspergillus is a ubiquitous soil‐dwelling organism that is found in humid areas, damp soil, agricultural environments, organic decay or decomposing matter.^1^ The risks of exposure vary both temporally and geographically and are dependent on precipitation patterns like air flow, humidity and temperature.^2^ Aspergillus can cause a variety of clinical syndromes ranging from mild, transient asthma to serious, disseminated disease, particularly in an immune‐suppressed host.^1^

## 2 CASE DISCUSSION

A 55‐years‐old female patient came with a chief complaint of cough and gradual weight loss for about 2 months. She also complained of difficulty in breathing and hemoptysis occasionally.

Laboratory investigations showed low hemoglobin 10.7 gm %, total leukocyte count of 8600/cu mm with a differential count of neutrophil 57%, lymphocyte 30%, eosinophils 11% and monocyte 02%. Platelets were within normal limit. Her HIV, HbsAg and anti HCV tests were non‐reactive.

**FIGURE 1:**
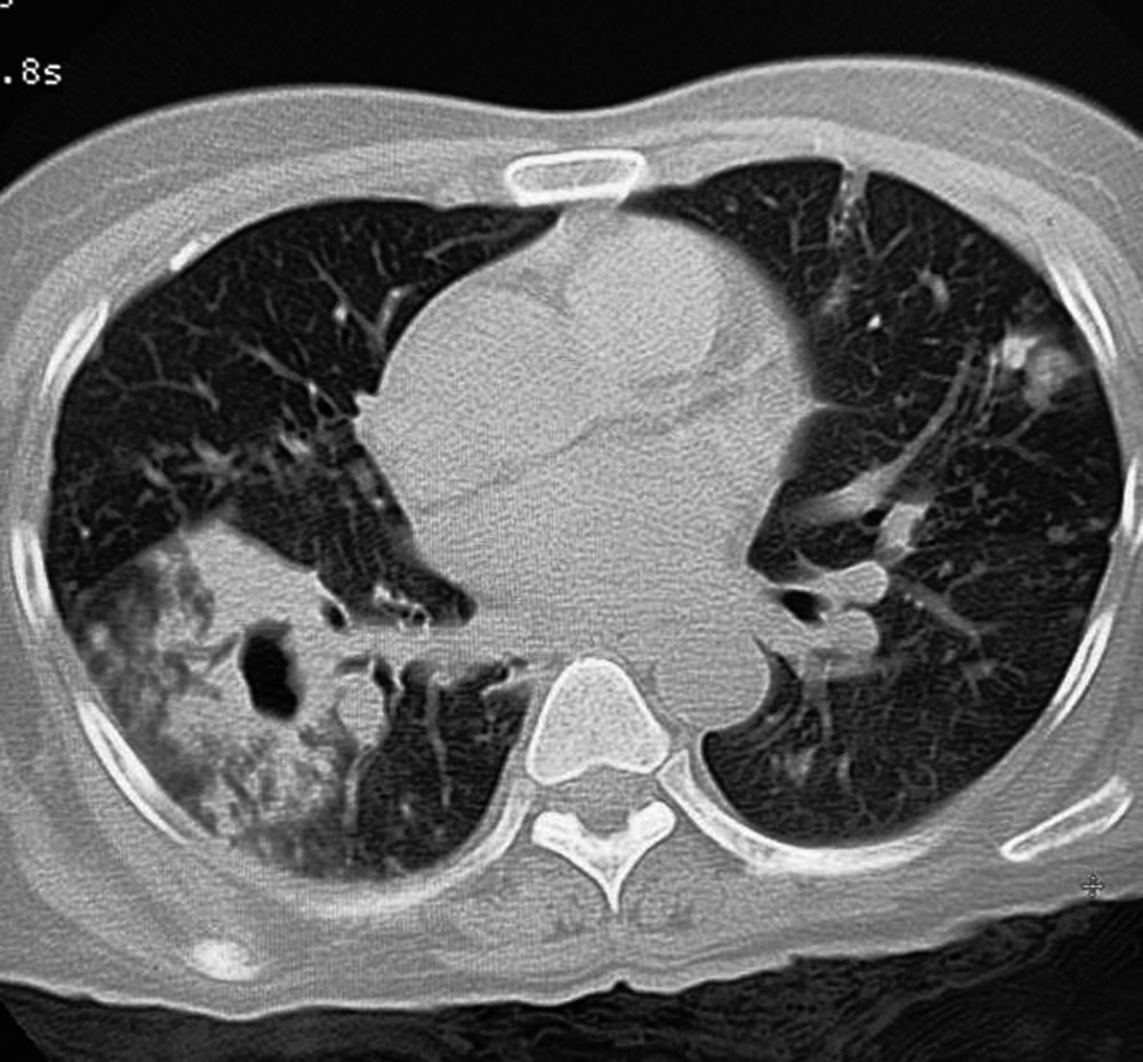
CT chest axial section, in lung window showing cavitation with surrounding consolidation in right lower lobe

**FIGURE 2:**
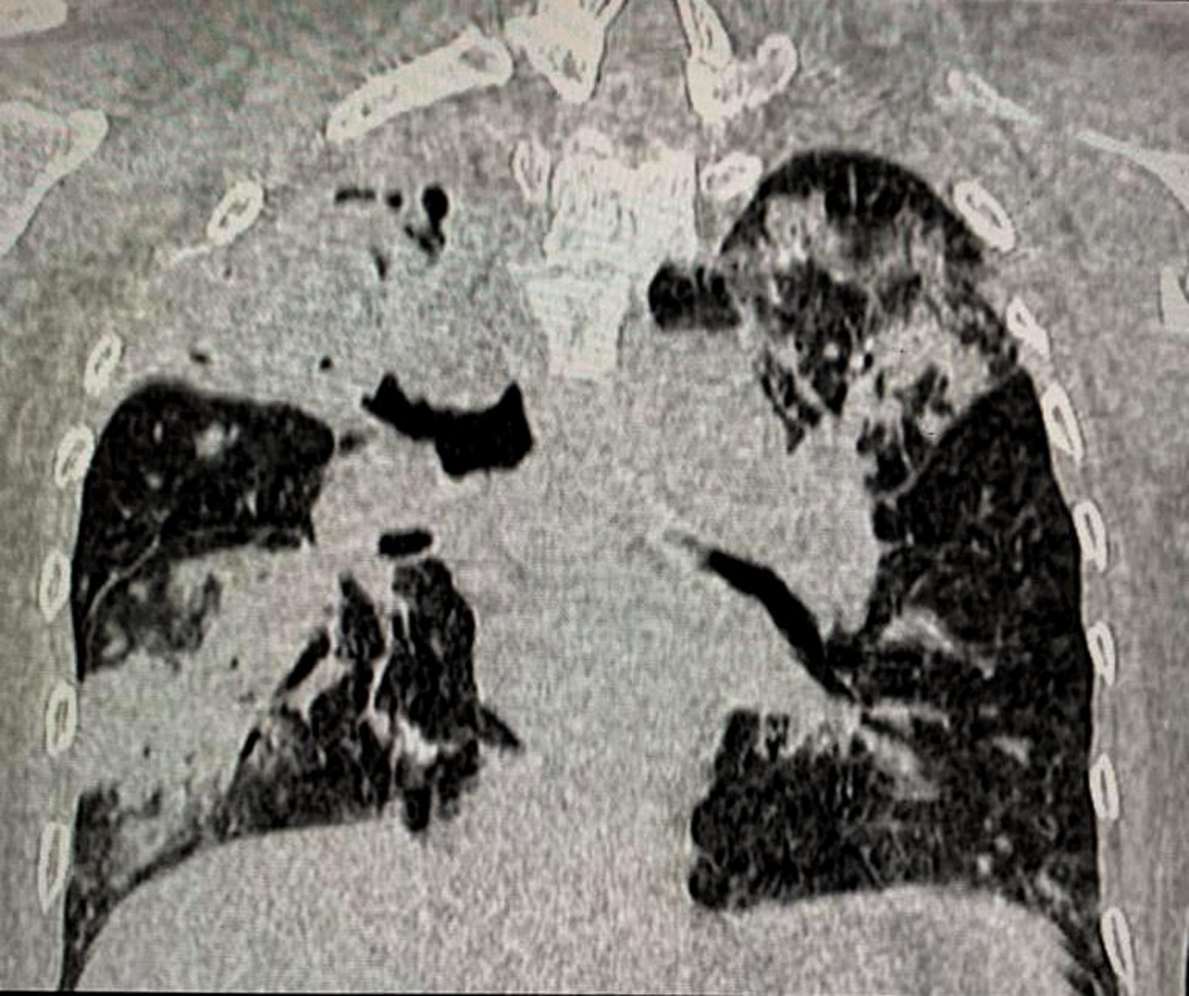
Coronal reformatted image in lung window showing consolidations in bilateral lungs

**FIGURE 3:**
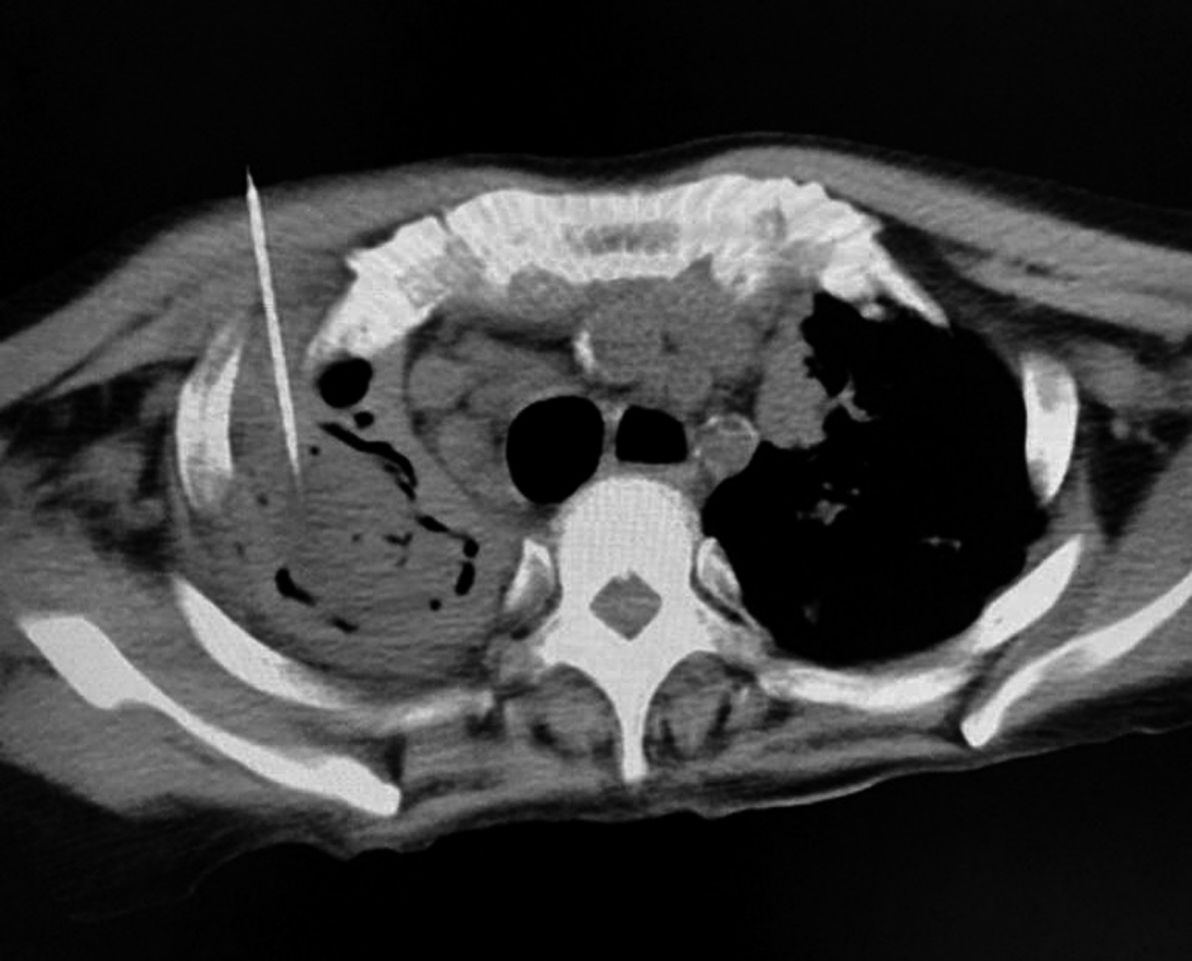
Image showing CT guided FNAC from right upper lobe lesion

Contrast enhanced computed tomography (CECT) of lung demonstrated well‐defined round‐to‐oval hypodense lesion with air pockets in right upper lobe measuring approximately 38.8 × 27.9 × 35.1 mm. Cavitary lesion with surrounding consolidation in right lower lobe (Figure 1) was present. Patchy consolidations with some lesions showing cavitations in left upper lobe of lung. Surrrounding reticulo‐nodular opacities were also recognized (Figure 2). Multiple enlarged mediastinal nodes were also notable. Radiological impression was suggestive of pulmonary Koch's with lung abscess. Patient was referred for drainage of abscess. Under all aseptic precautions, local anesthesia and CT guidance 18 gauze spinal needle were inserted (Figure 3). Significant purulent material could not be withdrawn on aspiration as suspected on CECT. Hence, aspiration was performed and slides were prepared for cytology.

**FIGURE 4:**
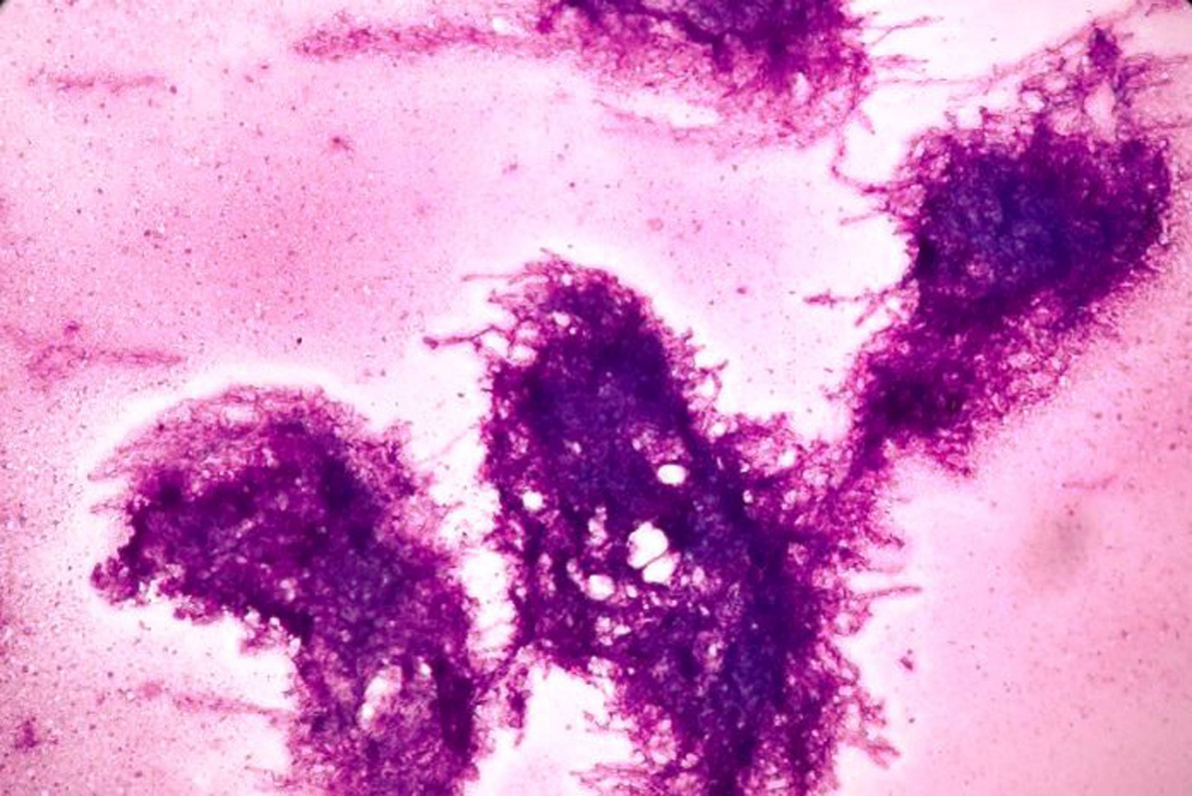
Cytology image showing several clusters of aspergillus hyphae. [Giemsa stain, 100x]

**FIGURE 5:**
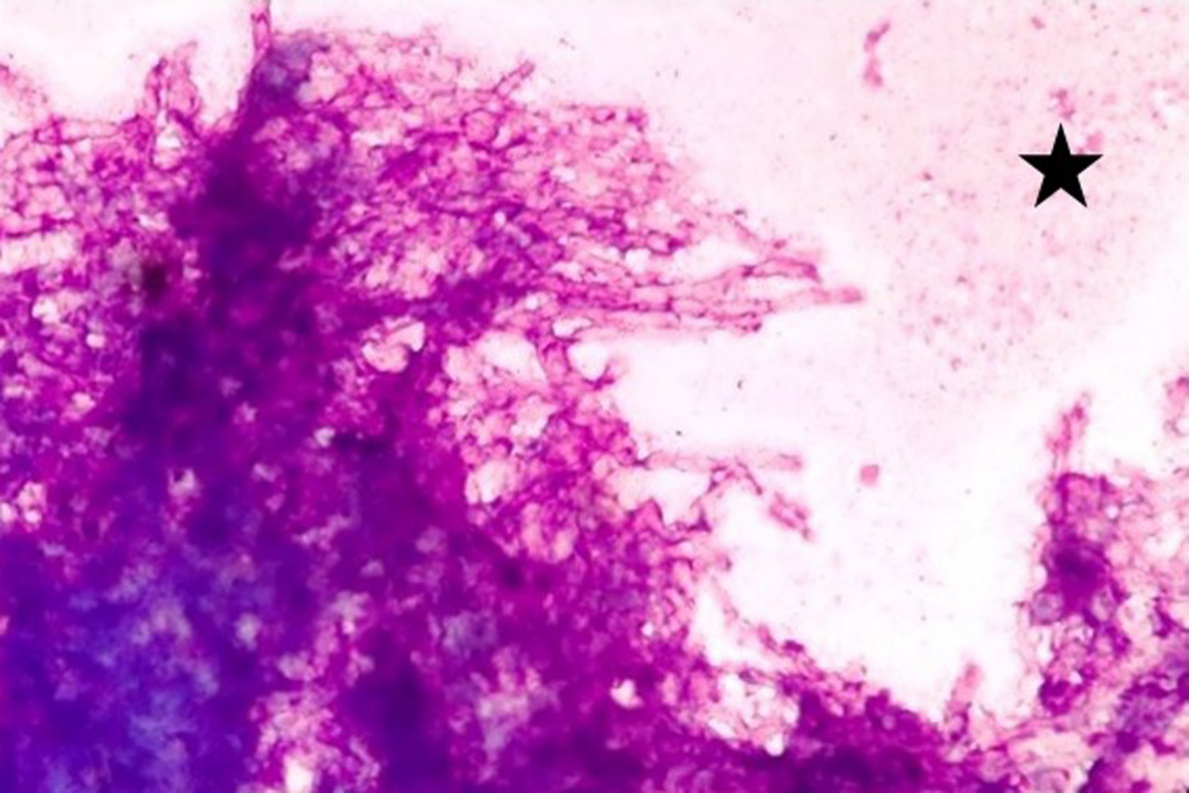
Cytology image showing aspergillus hyphae with septation and acute angle branching mimicking an arborizing tree along with areas of necrosis (asterix). [Giemsa stain, 400x]

Fine‐needle aspiration cytology showed mainly necrotic material with several clusters and balls of aspergillus (Figure 4) which demonstrated septate, acute‐angle‐branched hyphae mimicking an arborizing tree (Figure 5). Ziehl‐Neelsen stain for acid fast bacilli was performed which was negative. Cytological impression was given as aspergilloma of right lung.

Patient was treated with antifungal drug voriconazole for 6 months and on follow‐up, she was feeling better and didn't complain of cough, difficulty in breathing or hemoptysis.

## 3 DISCUSSION

Micheli in 1729 described a fungus as Aspergillus (rough head) because of the microscopic appearance of the spore‐bearing structure. The first recognized infection in man due to the fungus of this genus was described by Sluyter in 1847.^3^ There are approximately 300 species of the genus aspergillus. They are within the environment, but only, approximately, 8 species are responsible for the vast majority of human disease. Aspergillus fumigatus is the most common pathogen accounting for most of the infections followed by aspergillus niger.^4^

Aspergillus species can cause various forms of lung diseases like allergic bronchopulmonary aspergillosis which is a hypersensitivity reaction to aspergillus antigens mostly due to aspergillus fumigatus. The incidence of allergic bronchopulmonary aspergillosis varies from 6% to 20% of all patients with asthma.^5^ Most patients are under the age of 35 years at the time of diagnosis.^1^ Invasive pulmonary aspergillosis is another form of pulmonary disease which is characterized by proliferation of fungal mycelia in the pulmonary parenchyma. This disease is uncommon and is due to tissue invasion with the fungi.^1^ Recently, it has increased due to growing numbers of patients with impaired immune status associated with malignancy, organ transplantation and autoimmune conditions.^6^ Factors that predispose to the development of invasive aspergillosis include neutropenia, prolonged and high‐dose corticosteroid therapy, advanced AIDS and chronic granulomatous disease.^1^ Chronic necrotizing pulmonary aspergillosis is caused by aspergillus species which is associated with cavitary infiltrates in chronic lung disease or in mild immunodeficiency state.^6,7^ This infection causes progressive damage to the lung parenchyma without clear evidence of tissue invasion. The patients are usually middle‐aged with evidence of generalized immunosuppression in the form of diabetes mellitus, malnutrition, corticosteroid or radiation therapy.^1^ When aspergillus colonizes in a pre‐existing lung cavity, a fungus ball comprises of fungal hyphae, inflammatory cells, fibrin, mucus and tissue debris to form an aspergilloma.^1^ Pulmonary aspergilloma develops in pre‐existing lung cavities most commonly in patients with prior pulmonary tuberculosis but also in patients with other conditions as sarcoidosis and idiopathic pulmonary fibrosis.^8^ In the present case also, the patient had cavitary lesions due to past history of pulmonary tuberculosis.

Cysts and cavities are commonly encountered lesions in the lung on chest radiography and computed tomography (CT). Solid contents within a cavity may be seen in infectious processes, such as aspergillosis and in necrotic cancer. CT can show the size, shape and precise position of cysts and cavities when these details are not apparent on chest radiography.^7^

The hyphae of aspergillus species range in diameter from 2.5 to 4.5 µm and exhibit frequent septation. Aspergillus hyphae tend to branch dichotomously, progressively and primarily at acute angles of approximately 45°, mimicking an arborizing tree branch. Sometimes, in the areas of mycelial growth, hyphae often become tangled, bulbous and distorted which may cause difficulty in identification and diagnosis.

Fine‐needle aspiration cytology can be quite helpful in distinguishing malignancy from infection.^9^ This technique helps in detecting a wide variety of opportunistic pulmonary infections in immunocompromised patients.^10^ Fungal elements are detected using routine and special stains like Periodic Acid‐Schiff.^9^ This procedure is safe, cost‐effective and provides rapid results.^10^

Treatment of aspergilloma is considered when patients become symptomatic, usually with hemoptysis. Surgical resection is curative but may not be possible in patients with limited pulmonary function. Oral itraconazole may provide partial or complete resolution of aspergillomas in 60% of patients. In the present case, oral anti‐fungal drug was given for 6 months. Successful intracavitary treatment using CT guided percutaneous catheter instillation of amphotericin has been reported in small numbers of patients.^8^

## 4 CONCLUSION

Fine‐needle aspiration cytology is a simple, inexpensive and well‐established diagnostic technique which aid in early diagnosis and appropriate management of the patients with aspergilloma. This procedure also helps to rule out other differential diagnosis like neoplasms and inflammatory conditions.

## AUTHOR CONTRIBUTIONS

D.G.M – Study concept and design and paper writing. M.K.S – Conception of work and providing radiological images. A.G – Paper editing and guidance. D.U – Data collection and editing.

## CONFLICT OF INTEREST

The authors declare no conflict of interest.

## ETHICAL APPROVAL

Ethical approval is not required for case report publication.

## CONSENT

Written informed consent was obtained from the patient to publish this report in accordance with the journal's patient consent policy.
